# Novel antimicrobial and bioactive resin-based clear aligner attachment orthodontic materials

**DOI:** 10.3389/froh.2025.1630019

**Published:** 2025-08-07

**Authors:** Heba Alqarni, Ibrahim Ba-Armah, Nader Almutairi, Mohammad Alenizy, Dwayne D. Arola, Thomas W. Oates, Jirun Sun, Michael D. Weir, Hockin H. K. Xu

**Affiliations:** ^1^ Dental Biomedical Sciences PhD Program, Graduate School, University of Maryland, Baltimore, MD, United States; ^2^ Department of Biomaterials and Regenerative Dental Medicine, University of Maryland School of Dentistry, Baltimore, MD, United States; ^3^ Department of Pediatric Dentistry and Orthodontics Sciences, College of Dentistry, King Khalid University, Abha, Saudi Arabia; ^4^ Department of Restorative Dental Science, College of Dentistry, Imam Abdulrahman Bin Faisal University, Dammam, Saudi Arabia; ^5^ Department of Conservative Dental Sciences, College of Dentistry, Prince Sattam bin Abdulaziz University, Al-Kharj, Saudi Arabia; ^6^ Department of Restorative Dental Sciences, University of Hail, Hail, Saudi Arabia; ^7^ Materials Science and Engineering (MSE), University of Washington, Seattle, WA, United States; ^8^ Department of Advanced Oral Sciences and Therapeutics, University of Maryland, School of Dentistry, Baltimore, MD, United States; ^9^ Mineralized Tissue Biology Department, The ADA Forsyth Institute, Cambridge, MA, United States; ^10^ Center for Stem Cell Biology & Regenerative Medicine, University of Maryland School of Medicine, Baltimore, MD, United States; ^11^ Marlene and Stewart Greenebaum Cancer Center, University of Maryland School of Medicine, Baltimore, MD, United States

**Keywords:** dental orthodontics, antibacterial, nanoparticles, clear aligner attachment, white-spot lesions, bioactive and therapeutic

## Abstract

**Introduction:**

Clear aligner orthodontic treatment provides a hygienic and esthetic alternative to fixed appliances; however, the required resin attachments can promote plaque accumulation and increase the risk of white-spot lesions in enamel. This study aimed to develop a novel resin-based antibacterial and bioactive orthodontic clear aligner attachment and evaluate its mechanical and antibacterial properties.

**Methods:**

A resin matrix composed of urethane dimethacrylate (UDMA) and triethylene glycol divinylbenzyl ether (TEG-DVBE) was modified with 3% dimethylaminododecyl methacrylate (DMADDM) for antibacterial effects and nano-amorphous calcium phosphate (NACP) to support remineralization. Transbond™ LV and Vitremer™ were selected as commercial controls. Mechanical properties (flexural strength, elastic modulus, microhardness, and shear bond strength), degree of conversion, and antibacterial performance against *Streptococcus mutans (S. mutans)* biofilms were assessed through colony forming units (CFU), biofilm metabolic activity (MTT), and lactic acid production.

**Results:**

All experimental groups showed flexural strength of 100.6–109.2 MPa, exceeding the ISO standard for resin-based materials. Degree of conversion in experimental groups ranged from (53.4 ± 2.3 to 69 ± 0.9) %, significantly exceeding (47.5 ± 0.1) % for Transbond control (*p* < 0.05). Hardness was (0.21 ± 0.03) GPa for Transbond control, statistically comparable to (0.20 ± 0.02) GPa for the 20% NACP + 45% glass group. All experimental groups achieved a 6-log reduction in biofilm CFU and 90% reduction in metabolic activity and lactic acid production vs. controls (*p* < 0.01).

**Conclusion:**

This novel clear aligner attachment resin exhibits promising mechanical strength, high degree of conversion, potent antibacterial effects, and ion releases to potentially reduce white-spot lesions during clear aligner treatment.

## 1 Introduction

Orthodontic treatment has traditionally relied on fixed appliances such as brackets and wires to achieve desired tooth movement and alignment (1). While effective, these conventional systems are often associated with discomfort, esthetic concerns, and challenges in maintaining proper oral hygiene (2). In contrast, clear aligner therapy (CAT) is a modern alternative that addresses many of these limitations (3). Clear aligners are removable, transparent trays that gradually shift teeth into their desired positions, offering improved patient comfort and discretion (4). As technology and clinical techniques have advanced, the use of clear aligners has increased substantially in orthodontic practice. Recent data indicate that clear aligners now account for approximately 30%–45% of all orthodontic cases, reflecting a major shift in treatment preferences (5). This is largely driven by the aesthetic appeal of aligners and their ease of use during treatment (6).

To further enhance the effectiveness of clear aligner systems, especially in achieving precise and controlled tooth movement, auxiliary components known as attachments are commonly used (7). Attachments are small, tooth-colored or translucent structures made of resin-based composite materials, bonded directly to the enamel surface (8). Their primary function is to improve the aligner's grip, increase the surface area for force transmission and support the aligner's programmed movement (9). Typically fabricated from restorative composite resins, they are selected for their durability, bonding strength, and esthetic compatibility (10).

While attachments improve the biomechanics of clear aligner therapy, they may also present oral hygiene challenges. Resin-based materials bonded to enamel create plaque-retentive areas, increasing the risk of bacterial colonization and white-spot lesions (WSLs). Studies report a WSLs incidence of 2.85%–8.25% during clear aligner therapy (11). Prolonged and daily wear of aligners up to 22 h can reduce salivary flow, limiting its natural cleansing effect and promoting plaque buildup (12). These concerns highlight the need to improve attachment materials to reduce bacterial activity and protect the enamel surface.

Previously, antimicrobial and remineralizing agents have been studied mainly on fixed orthodontics adhesives, but no studies have focused on their applications in clear aligner attachment materials (13, 14). Resin-based dental materials incorporating antibacterial monomers such as dimethylaminododecyl methacrylate (DMADDM) have demonstrated long-term antibacterial properties without compromising mechanical characteristics and biocompatibility (15, 16).

DMADDM exerts its antibacterial effect through a contact-killing mechanism, where the positively charged quaternary amine interacts with the negatively charged bacterial cell membrane. This interaction disrupts the membrane's electrical stability, leading to osmotic rupture, membrane breakdown and bacterial death. Studies have validated that quaternary ammonium compounds (QAMs) disrupt both outer and cytoplasmic membranes, leading to cell lysis (17). A recent study has shown that the addition of 3% DMADDM to the resin-based composite provided potent antibacterial activity against *Streptococcus mutans* (*S. mutans)* biofilms while maintaining good mechanical qualities, and a higher degree of conversion (18).

In addition to antibacterial agents, nano-sized amorphous calcium phosphate (NACP) has shown great potential in dental materials due to its ability to repair demineralized tooth structures. NACP works by gradually releasing calcium and phosphate ions to restore the lost minerals in enamel and dentin (19). This mechanism is especially beneficial under acidic oral conditions. NACP-containing materials can be recharged, allowing them to absorb and release ions repeatedly, which supports extended protection over time (20).

However, a literature search revealed no report on the use of DMADDM, urethane dimethacrylate (UDMA) and triethylene glycol divinylbenzyl ether (TEGDVBE) to formulate a clear aligner attachment material for orthodontic applications. UDMA is widely studied in dental resins due to its low polymerization shrinkage, high mechanical strength, and favorable biocompatibility compared to Bis-GMA-based systems (21). TEG-DVBE is a hydrophobic ether-based diluent monomer, has been shown to improve resistance to hydrolytic and enzymatic degradation when incorporated into resin matrices (22). Therefore, developing such a bioactive resin incorporating these components may enable bacterial inhibition and white-spot lesions prevention during clear aligner orthodontic treatments.

The objectives of this study were to: (1) develop a novel antibacterial resin-based clear aligner attachment orthodontic material to inhibit the biofilm activity and reduce white-spot lesions; (2) investigate the effects of NACP and glass filler levels. The following hypotheses were tested: (1) The new antibacterial resin-based clear aligner attachment orthodontic material would substantially inhibit the activity of *S. mutans* biofilms, compared to the commercial controls; (2) incorporating 3% DMADDM with NACP and glass fillers into the UDMA/TEGDVBE resin would not adversely affect the mechanical properties, compared to commercial controls. The null hypotheses were: (1) The new antibacterial resin-based clear aligner attachment orthodontic material would have *S. mutans* biofilm activity similar to commercial controls; (2) incorporating 3% DMADDM with NACP and glass fillers into the UDMA/TEGDVBE resin would lower the mechanical properties, compared to commercial control.

## 2 Materials and methods

### 2.1 Formulation of resin-based attachment materials

Experimental light-cured orthodontic attachment composite was formulated using a mass fraction of 55.8% UDMA (Esstech, Essington, PA, USA) and 44.2% (TEG-DVBE). TEG-DVBE was produced by gradually adding triethylene glycol in dimethylformamide (DMF) to a stirred mixture of NaH in a temperature range of 0°C to 4°C under an argon ambiance for 30 min. Following two hours of agitation, 4-vinyl benzyl chloride in DMF was gradually added over 30 min. The resulting mixture was then agitated at ambient temperature for 18 h. Subsequently, the reaction mixture was neutralized by adding a saturated NH₄Cl solution (0.6 g/ml of water). The resultant solution was diluted with distilled water and extracted using ethyl acetate. The solvent was evaporated under vacuum, resulting in the formation of a light-colored oil (23). 0.2% camphorquinone (CQ) and 0.8% ethyl 4-N,N-dimethylaminobenzoate (4EDMAB) (MilliporeSigma, Burlington, MA, USA) were added as photoinitiators. This mixture is denoted as UV.

The antibacterial monomer DMADDM was synthesized via a modified Menschutkin reaction as described previously (13, 24). 10 mmol of 1-bromododecane (BDD, TCI America, Portland, OR, USA) and 10 mmol 2-(dimethylamino) ethyl methacrylate (DMAEMA, Sigma-Aldrich, Saint Louis, MO, USA) were mixed in 3 g of ethanol at 70°C for 24 h, followed by ethanol evaporation to yield a waxy solid mixture. DMADDM was incorporated into the UV resin at 3% by mass. The amount was selected following previous studies that demonstrated potent antibacterial activities at this percentage (16, 18).

Nanoparticles of amorphous calcium phosphate (NACP) (particle size = 116 nm) were synthesized using a spray-drying technique, as reported previously (19, 25). The NACP was added at two levels of 10% and 20% by mass, as they were shown to provide significant calcium and phosphate ion release for enamel remineralization, while maintaining acceptable mechanical properties (18, 25).

To reinforce the attachment composite mechanically, silanized barium boroaluminosilicate glass particles with a median size of 1.4 µm (Dentsply Sirona, Milford, DE, USA) were added as co-fillers. The three glass filler levels (45%, 50%, and 55%) were selected because glass filler levels lower than 45% compromised the composite mechanical properties. Glass filler levels greater than 55%, together with the NACP fillers, yielded a composite paste that was too dry to be mixed cohesively. These filler levels were also consistent with previous studies (16, 18, 25). Two commercial controls were selected: (1) Transbond™ Supreme LV (3M Unitek, Monrovia, CA, USA) is a light-cure orthodontic adhesive composed mainly of Bis-GMA and glass fillers, widely used as an attachment material for clear aligner orthodontics. and (2) Vitremer™ (3M ESPE, St. Paul, MN, USA) is a dual-cure resin-modified glass ionomer, with fluoride-releasing capability. The experimental groups were formulated based on the previously reported resin-based dental materials, eliminating the need for additional experimental control (16).

A 2 × 3 full-factorial design was used to evaluate the effect of different component levels, with two levels of NACP (10%, 20%), and three levels of glass filler level (45%, 50%, 55%), producing the following six formulations:

(1) UV + 3% DMADDM + 10% NACP + 45% glass
(2) UV + 3% DMADDM + 10% NACP + 50% glass
(3) UV + 3% DMADDM + 10% NACP + 55% glass
(4) UV + 3% DMADDM + 20% NACP + 45% glass
(5) UV + 3% DMADDM + 20% NACP + 50% glass
(6) UV + 3% DMADDM + 20% NACP + 55% glass

Preliminary testing showed that the two pastes of UV + 3% DMADDM + 20% NACP + 50% glass and UV + 3% DMADDM + 20% NACP + 55% glass were too dry to be mixed cohesively. Therefore, these two groups were excluded from further testing. The following Table 1 shows the final experimental and control groups:

**Table 1:** Compositions of the control and experimental resin-based clear aligner attachment groups.

Group name	Compositions
Commercial control 1: Transbond™ Supreme LV (3M Unitek, Monrovia, CA) referred to as Transbond Control	TEGDMA, Bis-GMA, BISEMA, silane-treated zirconia, ceramic and silica, reacted polycaprolactone polymer, diphenyliodonium hexafluorophosphate, and N, N-dimethylbenzocaine
Commercial control 2: Vitremer™ (3M ESPE, St. Paul, MN, USA) referred to as Vitremer Control	Fluoroaluminosilicate glass, polyalkenoic acid, 2-Hydroxyethyl methacrylate (HEMA), photoinitiators, and water
Experimental group 1 (referred to as 3DMADDM + 10NACP + 45glass)	UV (53.55% UDMA + 43.45% TEG-DVBE) + 3% DMADDM + 10% NACP + 45% glass
Experimental group 2 (referred to as 3DMADDM + 10NACP + 50glass)	UV (53.55% UDMA + 43.45% TEG-DVBE) + 3% DMADDM + 10% NACP + 50% glass
Experimental group 3 (referred to as 3DMADDM + 10NACP + 55glass)	UV (53.55% UDMA + 43.45% TEG-DVBE) + 3% DMADDM + 10% NACP + 55% glass
Experimental group 4 (referred to as 3DMADDM + 20NACP + 45glass)	UV (53.55% UDMA + 43.45% TEG-DVBE) + 3% DMADDM + 20% NACP + 45% glass

### 2.2 Mechanical properties

Orthodontic aligner resin attachment specimens were fabricated using 2 × 2 × 25 mm^3^ stainless-steel mold. Specimens were covered with Mylar strips and then photo-cured for 60 s per side at 1,200 mW/cm^2^ using a Labolight DUO curing device (GC, Tokyo, Japan). Before testing, samples were stored at 37°C for 24 h. A three-point flexural test (*n* = 6) was conducted using a computer-controlled Universal Testing Machine (Insight 1, MTS, Cary, NC, USA) with a span of 10 mm and a crosshead speed of 1 mm/min to measure the flexural strength and elastic modulus. Flexural strength was calculated as: FS = (3 × *P*_max_ × *L*)/(2 × *b* × *h*^2^) where *P*_max_ is the maximum load at fracture, *L* is the span length, *b* is the specimen width, and *h* is the specimen thickness. Elastic modulus was calculated as: *E* = (*P*/*d*) × (*L*^3^)/(4 × *b* × *h*^3^) where *P*/*d* is the slope of the load–deflection curve in the linear elastic region. The test was conducted following ISO 4049:2019 protocols (18, 24, 26).

### 2.3 Degree of polymerizations conversion

The degree of conversion (DC) of orthodontic aligner attachment resin materials was measured using Fourier-transform infrared spectroscopy (FTIR-ATR) (Nicolet 6700, Thermo Fisher Scientific, Waltham, MA, USA). Samples (*n* = 3) were standardized to a 1 mm thickness and analyzed over the 400–4,000 cm^−1^ range with 32 scans and a resolution of 4 cm^−1^. The DC was calculated by comparing the intensity of the aliphatic *C* = *C* peak at 1,637 cm^−1^ before and after light curing, using the aromatic *C* = *C* peak at 1,583 cm^−1^ as an internal reference as previously described (27, 28). Light polymerization was performed for 40 s using a Labolight DUO curing device (GC, Tokyo, Japan) with an intensity ≥1,000 mW/cm^2^. For the Vitremer control group, which is a dual-cure material, the DC was assessed using the internal reference peak at 1720 cm^−1^, with measurements taken after 24 h to account for its chemical curing component (29). The DC values were calculated using the following equation.

$$\mathrm{DC}(\text{\%})\;=\;\left(1-\frac{{\left(A_{1637}/A_{1583}\right)}_{\mathrm{post}\;\mathrm{cure}}}{{\left(A_{1637}/A_{1583}\right)}_{\mathrm{before}\;\mathrm{cure}}}\right)\;\times 100$$

### 2.4 Enamel—attachment shear bond testing

A total of 90 extracted human premolars were randomly distributed among the experimental groups (*n* = 15). To provide a flat bonding surface, each tooth was sectioned sagittally using a trim saw with a diamond blade (Lapcraft Trim Saw; Lapcraft, Powell, OH, USA). The sectioned specimens were then embedded in self-curing acrylic resin (Lang Dental Manufacturing, Wheeling, IL, USA) to ensure stability and proper orientation during testing.

The enamel coronal and buccal surfaces were cleaned with oil-free pumice using a rubber cup at low speed for 10 s, then rinsed and dried for 15 s, etched with 35% phosphoric acid (Scotchbond, 3M ESPE, St. Paul, MN, USA) for 30 s, then rinsed and dried again as instructed by the manufacturer. After that, each group was bonded to the enamel surface using a bonding clamp assembled with a mold insert (Model no. 34,224 and 34,228; Ultradent, South Jordan, UT, USA), then light-cured for 60 s using a curing unit (Optilux VCL 401, Demetron Kerr, Danbury, CT, USA). Samples were stored in distilled water at 37°C for 24 h before testing. Shear bond strength was tested using a Universal Testing Machine (Insight 1, MTS, Cary, NC, USA) attached to a chisel-shaped loading blade. A vertical force was applied at a load of 1kN and speed of 0.5 mm/min until the resin attachments were debonded from the specimens. The shear bond strength values (MPa) were obtained by dividing the peak load at failure (*N*) by the adhesive/attachment base surface area (mm^2^). This method followed previously reported testing protocols (13, 30).

### 2.5 Vickers hardness test

The surface hardness of the orthodontic aligner attachment resin specimens was evaluated using the Vickers microhardness test. Rectangular resin bars at 2 × 2 × 12 mm dimensions for each group samples (*n* = 10) were fabricated and stored in distilled water at 37°C for 24 h before testing. Microhardness measurements were performed using a Vickers microhardness tester (HMV II; Shimadzu Corporation, Kyoto, Japan), with a load of 980.7 mN applied for a dwell time of 10 s. Indentation lengths were measured using 10× or 20× objective lenses, depending on visibility. Four indentations were made on each specimen and then Vickers hardness number (VHN) was calculated by the device based on the diagonal lengths of the indentations, following standard methodology (31).

### 2.6 Resin-based attachment samples for biofilm testing

Disk-shaped specimens of the orthodontic aligner attachment resin (*n* = 6) were prepared with a diameter of 8 mm and a thickness of 1 mm. Each sample was light cured for 60 s on both sides using a Labolight DUO curing unit (GC, Tokyo, Japan) at an intensity of 1,200 mW/cm^2^, then stored at 37°C for 24 h to complete polymerization. To eliminate any residual unreacted monomers, the samples were immersed in distilled water and agitated at 100 rpm for one hour (26). Following this, the specimens were sterilized with ethylene oxide gas (Anprolene AN 74i, Andersen, Haw River, NC, USA) and degassed for seven days following the manufacturer's guidelines to ensure the removal of residual ethylene oxide (18, 26).

### 2.7 *Streptococcus mutans* biofilm model

Approval for the use of bacterial species in this study was obtained from the Institutional Review Board of the University of Maryland, Baltimore (HP-00052180). *S. mutans* (UA159) was selected due to its well-established role in dental caries. The bacteria were cultured in brain heart infusion (BHI) broth (Sigma-Aldrich, St. Louis, MO, USA) for 16–18 h at 37°C in 5% CO₂ prior to all biofilm assays. The bacterial inoculum was standardized to 10⁷ (CFU/ml) using a spectrophotometer (Genesys 10S, Thermo Scientific, Waltham, MA, USA), based on the standard curve of optical density at 600 nm (OD600) vs. CFU/ml. Each resin specimen was placed in the wells of a 24-well plate, containing 1.5 ml of BHI culture medium with 2% sucrose, and incubated for 24 h. Later, the samples were transferred to fresh 24-well plates containing 1.5 ml of new BHI media with sucrose and incubated for an additional 24 h, allowing the development of mature biofilms. The total incubation time of 48 h, consistent with previous studies (32).

### 2.8 Biofilm colony-forming unit (CFU) counts

After 48 h of biofilm development, the orthodontic aligner attachment resin discs (*n* = 6) were transferred to a 24-well plate containing 1 ml of phosphate-buffered saline (PBS). Biofilms were harvested from each specimen by scraping, sonication and vortexing (FS-30, Fisher Scientific, Pittsburgh, PA, USA) to ensure effective detachment of adherent bacteria. The resulting bacterial suspensions were then serially diluted (10^1^–10⁶-fold) and plated onto BHI agar plates. The plates were incubated at 37°C in 5% CO₂ for 48 h. After incubation, colony counts were performed using a Reichert Quebec Darkfield Colony Counter (Depew, NY, USA). The number of CFUs was calculated by multiplying the colony count by the corresponding dilution factor. Each CFU assay was conducted in triplicate to ensure accuracy and reproducibility (33).

### 2.9 Biofilm metabolic activity (MTT assay)

The metabolic activity of *S. mutans* biofilms was evaluated using a colorimetric assay with 3-[4,5-dimethylthiazol-2-yl]-2,5-diphenyltetrazolium bromide (MTT) (WST-8, Selleckchem, Houston, TX, USA). After 24 h of incubation, composite discs with mature biofilms (*n* = 6) were placed in 24-well plates containing 1 ml of MTT solution (0.5 mg/ml in PBS) and incubated at 37°C with 5% CO₂ for 1 h. Following incubation, the specimens were transferred to new 24-well plates filled with 1 ml of dimethyl sulfoxide (DMSO) to dissolve the formazan crystals and left in the dark at (25°C) for 20 min. Subsequently, 200 µl of the DMSO solution from each sample was transferred to a 96-well plate. Absorbance was measured at 540 nm using a microplate reader (SpectraMax M5, Molecular Devices, Sunnyvale, CA, USA). A higher absorbance value indicates greater metabolic activity of the biofilm. The experiment was performed in triplicate (15).

### 2.10 Lactic acid production by biofilms

After 48 h of biofilm development, the orthodontic aligner attachment resin samples (*n* = 6) were transferred to 24-well plates containing 1.5 ml of buffered peptone water (BPW; Aldrich, St. Louis, MO, USA) supplemented with 0.2% sucrose. The samples were incubated for 3 h at 37°C in a 5% CO₂ atmosphere. Following incubation, lactate production was quantified using an enzymatic lactate dehydrogenase assay, and the optical density was measured at 340 nm with a microplate reader (SpectraMax M5, Molecular Devices, Sunnyvale, CA, USA). Higher absorbance indicates increased lactic acid production by the biofilm. The experiment was performed in triplicate, following previously established protocols (15, 34).

### 2.11 Statistical analysis

All statistical analyses were performed using Sigma Plot software (SYSTAT, Chicago, IL, USA). Normality and power analyses were conducted before testing. Data were analyzed using one-way analysis of variance (ANOVA) followed by Tukey's *post hoc* test to determine statistically significant differences among groups. A *p*-value < 0.05 was considered statistically significant.

## 3 Results

### 3.1 Flexural strength and elastic modulus

**Figure 1:**
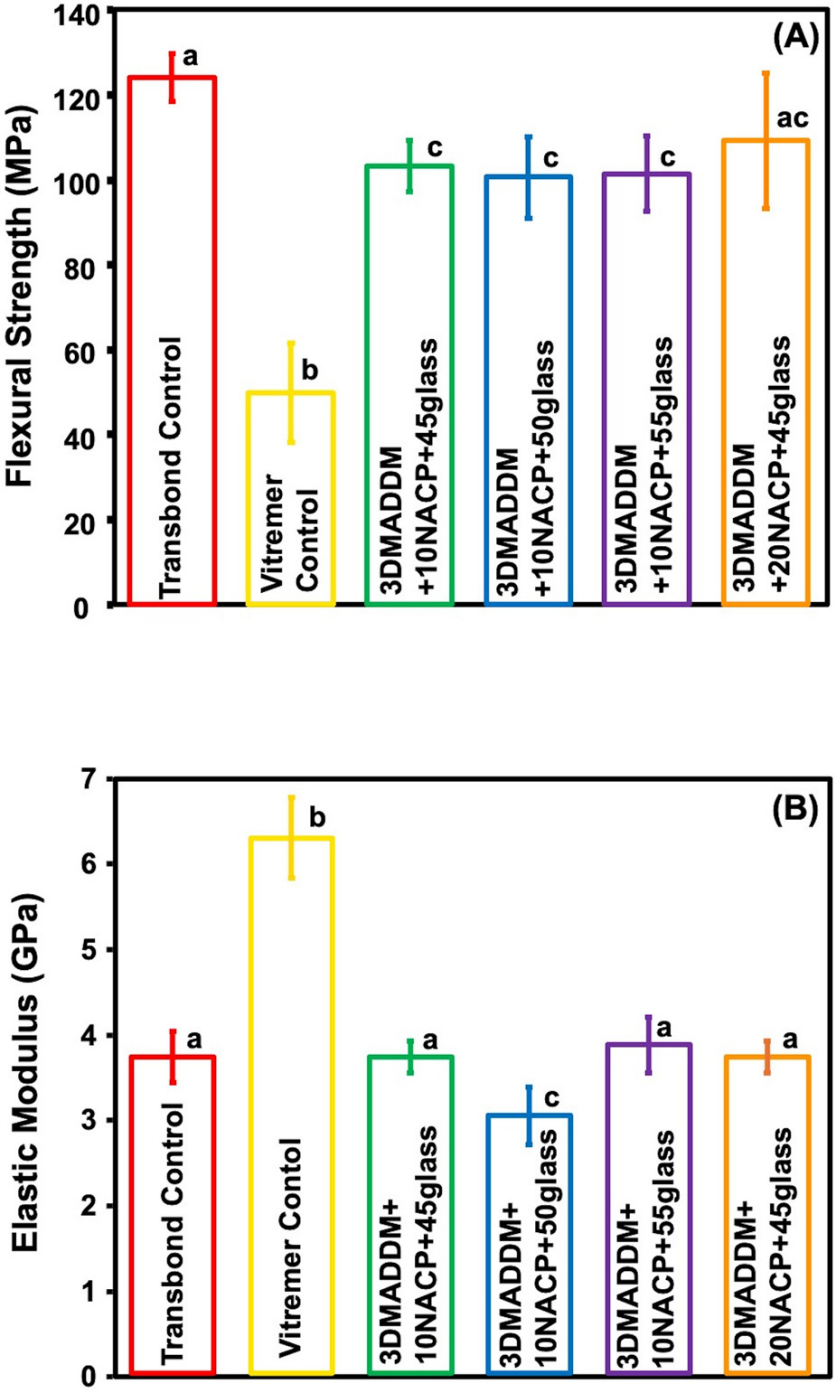
Mechanical properties of tested resin-based attachment materials: **(A)** flexural strength and **(B)** elastic modulus (mean ± SD; *n* = 6). The results demonstrated that incorporating NACP and glass along with 3% DMADDM did not compromise the mechanical properties. Experimental groups showed values exceeding ISO recommendations for resin-based materials. The elastic modulus of the experimental groups was comparable to that of Transbond control but lower than Vitremer control. The experimental group containing 10% NACP and 50% glass exhibited a lower elastic modulus compared to Transbond control and other experimental groups. Different letters indicate statistically significant differences among groups (*p* < 0.05).

The flexural strength values of the orthodontic aligner attachment resin material are presented in Figure 1A (mean ± SD; *n* = 6). Transbond control demonstrated flexural strength (124.0 ± 5.5) MPa, which was significantly greater than Vitremer control (49.7 ± 11.6) MPa (*p* < 0.01). Among the experimental groups, the formulation with 20% NACP + 45% glass showed a high flexural strength (109.2 ± 15.8) MPa, which was comparable to Transbond control but significantly higher than Vitremer control (*p* < 0.01).

The other experimental groups (10% NACP + 45% glass, 10% NACP + 50% glass, and 10% NACP + 55% glass) showed flexural strength values ranging from (103.1 ± 6.1) MPa to (100.5 ± 9.6) MPa, all of which were significantly higher than Vitremer control (*p* < 0.01) and exceeded the ISO standard (≥80 MPa) for resin-based materials. No statistically significant differences were observed among all the experimental groups (*p* > 0.05).

The elastic modulus values are shown in Figure 1B (mean ± SD; *n* = 6). Vitremer control exhibited the highest elastic modulus (6.2 ± 0.4) GPa, which was significantly greater than all other groups (*p* < 0.05). The elastic modulus of Transbond control and all experimental groups with 20% or 10% NACP with varying glass filler contents ranged between (3.7 ± 0.1) GPa and (3.8 ± 0.3) GPa. There were no significant differences among them (*p* > 0.05), except for the 10% NACP + 50% glass group, which showed a slightly lower value (3.0 ± 0.3) GPa and was significantly different when compared to the others (*p* < 0.05). Overall, incorporation of NACP and varying glass content did not substantially affect the elastic modulus, compared to Transbond control.

### 3.2 Degree of polymerization conversion

**Figure 2:**
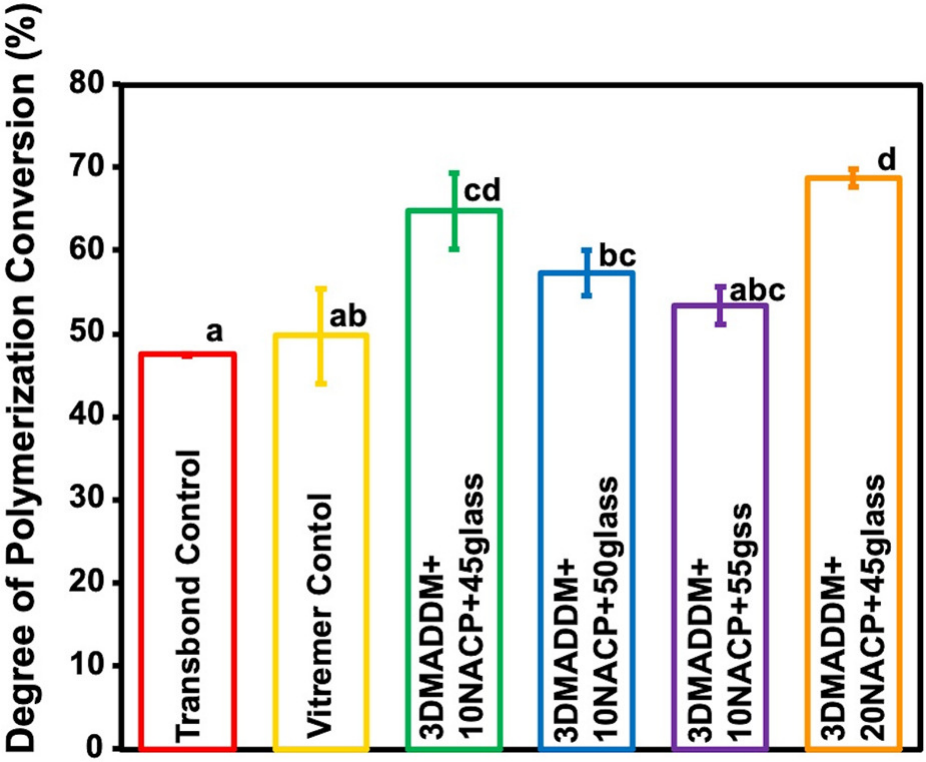
Degree of conversion of resin-based attachment materials (mean ± SD; *n* = 3). These results demonstrate that incorporating different mass fractions of NACP and glass, along with 3% DMADDM, resulted in increased degree of conversion values exceeding those of the commercial controls. Experimental groups 1 and 2 with 45% glass showed the highest polymerization rates compared to other experimental groups. Different letters indicate statistically significant differences among groups (*p* < 0.05).

The degree of conversion (DC) result is presented in Figure 2 (mean ± SD; *n* = 3). Transbond control showed the lowest DC (47.5 ± 0.1) %, significantly lower than 20% NACP + 45% glass (68.7 ± 0.9) %, *p* < 0.05), which exhibited the highest conversion. Both Transbond control and Vitremer control were significantly lower than the experimental groups (*p* < 0.05). All experimental groups demonstrated higher DC values compared to Transbond control and Vitremer control, with 20% NACP + 45% glass (68.7 ± 0.9) % being significantly higher than both controls but statistically similar to 10% NACP + 45% glass. The other experimental groups 10% NACP + 50% glass and 10% NACP + 55% glass showed DC values of (57.3 ± 2.7) % and (53.4 ± 2.3) %, respectively, significantly higher than Transbond control (*p* < 0.05), but lower than the other experimental groups.

### 3.3 Vickers hardness test

**Figure 3:**
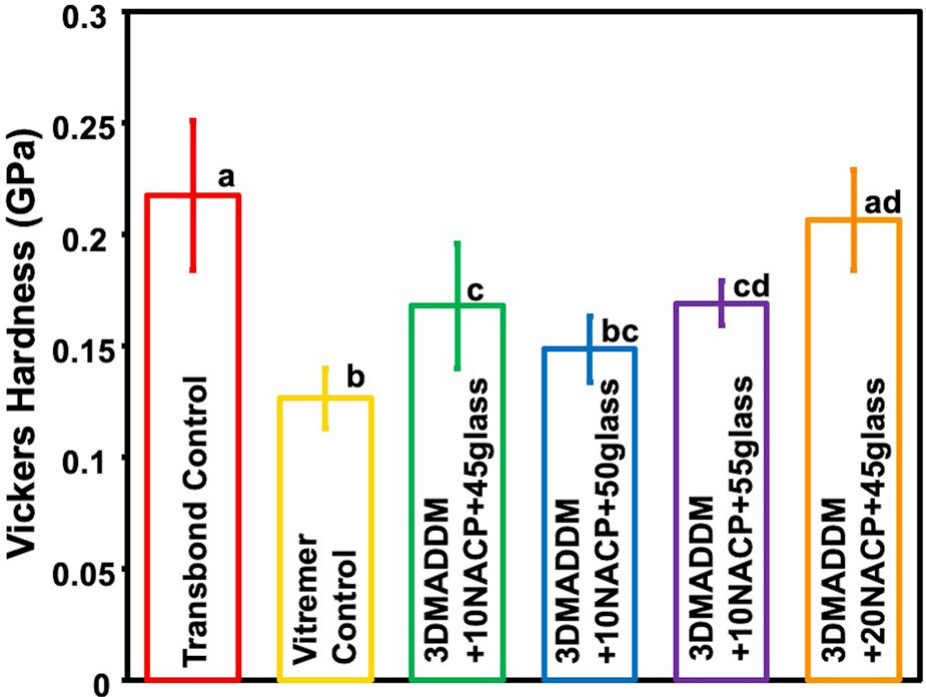
Vickers hardness of resin-based attachment materials (mean ± SD; *n* = 10). The results demonstrate that incorporating different mass fractions of NACP and glass, along with 3% DMADDM, influenced the surface hardness of the materials. The experimental group containing 20% NACP and 45% glass showed the highest hardness values compared to other experimental groups. Values with different letters are significantly different from each other (*p* < 0.05).

The Vickers hardness results are presented in Figure 3 (mean ± SD; *n* = 10). Transbond control showed the highest hardness value (0.21 ± 0.03) GPa, which was significantly greater than all other groups (*p* < 0.05). Vitremer control had the lowest hardness (0.12 ± 0.01) GPa, significantly lower than both 20% NACP + 45% glass (0.20 ± 0.02) GPa (*p* < 0.05) and 10% NACP + 55% glass (0.17 ± 0.009) GPa (*p* < 0.05). Among the experimental groups, 20% NACP + 45% glass showed the highest microhardness (0.20 ± 0.02) GPa, statistically similar to Transbond control, but significantly higher than the other experimental formulations (*p* < 0.05). Overall, incorporating NACP and glass fillers influenced the surface hardness of the orthodontic aligner attachment resin, with 20% NACP + 45% glass exhibited the most comparable performance to Transbond control.

### 3.4 Shear bond strength

**Figure 4:**
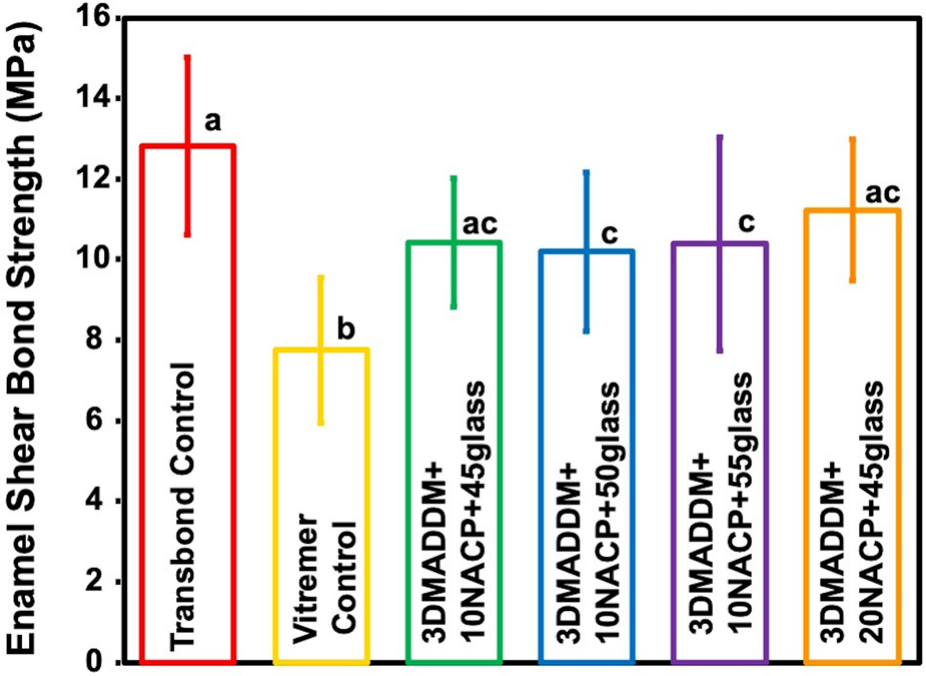
Shear bond strength to enamel of resin-based attachment materials (mean ± SD; *n* = 15). The results demonstrate that incorporating different mass fractions of NACP and glass, along with 3% DMADDM, maintained similar bond strength values. All experimental groups exhibited bond strengths comparable to Transbond control. Values with different letters are significantly different from each other (*p* < 0.05).

Shear bond strength values to enamel are shown in Figure 4 (mean ± SD; *n* = 15). Transbond control exhibited bond strength of (13 ± 2.2) MPa, which was significantly higher than Vitremer control (7.7 ± 1.8) MPa (*p* < 0.05). Among the experimental groups, 20% NACP + 45% glass (11.2 ± 1.7) MPa and 10% NACP + 45% glass (10.4 ± 1.6) MPa displayed comparable bond strengths to Transbond control, with no significant differences (*p* > 0.05). These findings indicate that incorporation of 3% DMADDM along with NACP and glass fillers can maintain adequate bonding performance to the enamel, with the highest bond strength achieved in formulations containing 20% NACP and 45% glass.

### 3.5 Colony-forming unit counts (CFU)

**Figure 5:**
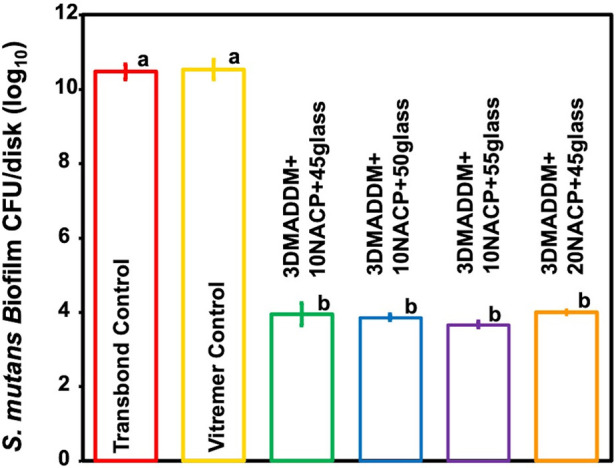
*S. mutans* biofilm colony-forming units (CFUs) per disk (log₁₀) (mean ± SD; *n* = 6) for Transbond control and Vitremer control, and experimental groups with UV + 3% DMADDM. All experimental groups showed a reduction of approximately 6 log₁₀ units in CFU counts. Groups with different letters indicate statistically significant differences (*p* < 0.05).

The CFU counts of 48 h *S. mutans* biofilms on the resin specimens are shown in Figure 5 (mean ± SD; *n* = 6). Both Transbond control and Vitremer control showed the highest biofilm accumulation, with values of (10.47 ± 0.21) and (10.52 ± 0.27) log CFU/disk, respectively. In contrast, all experimental groups containing 3% DMADDM exhibited a significant reduction in CFU counts, ranging from (3.67 ± 0.08) to (4.01 ± 0.05) log CFU/disk, representing a 6-log reduction compared to Transbond control and Vitremer control (*p* < 0.01). These results confirm that incorporating 3% DMAHDM into the orthodontic aligner attachment resin significantly inhibited *S. mutans* biofilm formation across all tested formulations.

### 3.6 Biofilm metabolic activity (MTT assay)

**Figure 6:**
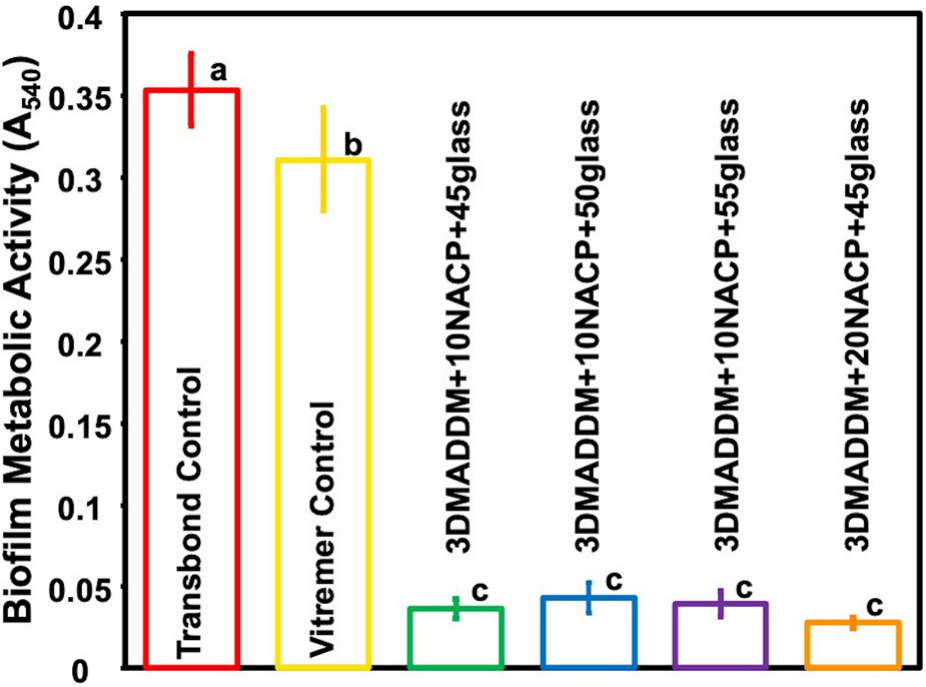
Biofilm metabolic activity of *S. mutans* on resin-based attachment materials (mean ± SD; *n* = 6) for Transbond control and Vitremer control, and experimental groups with UV + 3% DMADDM. All experimental groups showed a significant reduction in metabolic activity compared to Transbond control and Vitremer control. The experimental groups exhibited more than 90% reduction in biofilm metabolic activity. Groups with different letters indicate statistically significant differences (*p* < 0.05).

The metabolic activity of 48-hour *S. mutans* biofilms, assessed using the MTT assay, is presented in Figure 6 (mean ± SD; *n* = 6). Transbond control exhibited the highest metabolic activity (0.35 ± 0.02) A₅₄₀, followed by Vitremer control (0.31 ± 0.03) A₅₄₀. All experimental groups containing 3% DMADDM into the orthodontic aligner attachment resin demonstrated a significantly lower metabolic activities ranged from (0.03 ± 0.00) to (0.04 ± 0.01) A₅₄₀, showing around 90% reduction compared to Transbond control and Vitremer control (*p* < 0.01). No significant differences were observed among the experimental groups.

### 3.7 Biofilm lactic acid production

**Figure 7:**
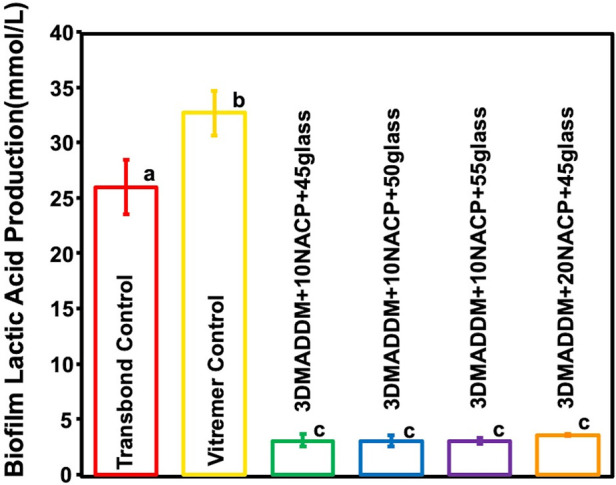
The concentration of lactic acid produced by *S. mutans* biofilm on resin attachment materials was assessed for the Transbond control and Vitremer control and experimental groups (mean ± SD; *n* = 6). All experimental groups containing 3% DMADDM demonstrated a significant reduction in lactic acid production, showing 90% reduction compared to Transbond control and Vitremer control. Different letters represent values that are significantly different from one another (*p* < 0.05).

Lactic acid production results are shown in Figure 7 (mean ± SD; *n* = 6). Vitremer control exhibited the highest lactic acid level (32.6 ± 2.04) mmol/L, significantly higher than Transbond control (26 mmol/L, *p* < 0.05). All experimental groups of the orthodontic aligner attachment resin showed significantly lower lactic acid levels (3.5 ± 0.05) mmol/L, indicating an approximate 90% reduction compared to Transbond control and Vitremer control (*p* < 0.01). No significant differences were observed among the experimental groups (*p* > 0.05), suggesting that all DMADDM-containing formulations were similarly effective in suppressing acidogenic activity.

## 4 Discussion

In this study, a novel resin-based clear aligner attachment orthodontic material was developed by combining a UDMA/TEGDVBE resin matrix with 3% DMADDM for antibacterial activity, nano-amorphous calcium phosphate (NACP) for remineralization, and glass fillers for mechanical reinforcement. Among the experimental groups, the formulation containing 20% NACP and 45% glass demonstrated the best antibacterial efficacy while maintaining desirable mechanical and physical properties.

This newly developed orthodontic aligner attachment resin material significantly reduced biofilm adhesion, with a 6-log reduction in CFU counts, a 95% decrease in metabolic activity, and a substantial reduction in lactic acid production. Simultaneously, it exhibited good shear bond strength and a higher degree of conversion. These results suggest that this bioactive and antibacterial resin-based attachment could play a crucial role in preventing white-spot lesions by inhibiting oral biofilm activity and potentially supporting the remineralization of demineralized enamel, contributing to improved dental care during clear aligner therapy.

The mechanical performance of attachment materials is critical for their clinical use, particularly in withstanding the forces during repeated placement and removal of thermoplastic aligners. In this study, all experimental groups exceeded the ISO standard flexural strength requirement (≥80 MPa), indicating sufficient structural integrity (35). The 20% NACP + 45% glass group showed flexural strength comparable to Transbond control and significantly higher than Vitremer control. The Vitremer control is a resin-modified glass ionomer with a highly cross-linked ionic structure and high glass filler content, which increases stiffness (high modulus) but makes it more brittle and prone to fracture under bending, resulting in lower flexural strength compared to resin-based composites (36). The higher flexural strength observed in the 20% NACP + 45% glass group compared to Control 2 could be primarily due to the reinforcement provided by the NACP nanoparticles and optimized glass filler ratio. Previous studies have shown that NACP can effectively enhance mechanical properties by providing improved stress distribution and increased filler-matrix bonding (37). Additionally, the carefully balanced ratio of glass fillers helps to maintain structural integrity without negatively impacting polymerization.

Elastic modulus values were generally comparable among experimental groups, except for a slight reduction in the 10% NACP + 50% glass group. Surface hardness remained favorable, with 20% NACP + 45% glass performing similarly to the Transbond control. Among the experimental groups, increasing the glass filler load did not improve flexural strength, elastic modulus, or hardness. Although higher filler content can initially enhance mechanical properties, exceeding the optimal level may result in a plateau or even reduced performance. This could be attributed to particle agglomeration, a reduced amount of resin matrix available for effective stress distribution, and weak bonding at the filler–resin interface (38, 39).

According to a previous study, a minimum shear bond strength (SBS) range of 6–8 MPa is required to withstand typical orthodontic forces (40). Our results showed that all experimental groups achieved SBS values above this clinically acceptable threshold, indicating adequate bonding performance for orthodontic clear aligner attachments. Notably, the group with 20% NACP + 45% glass and 10% NACP + 45% glass exhibited bond strength values comparable to the Transbond control, confirming that the incorporation of bioactive fillers and antibacterial agents did not compromise adhesive performance.

The degree of conversion (DC) is an essential factor influencing the performance and longevity of resin-based materials. A higher DC reflects more polymerization, resulting in better mechanical strength, lower monomer release, and improved biocompatibility. In this study, the incorporation of NACP and varying glass filler concentrations yielded DC values higher than the Transbond control and Vitremer control. Particularly, the 20% NACP + 45% glass group showed the highest DC among the experimental groups, indicating that the addition of bioactive fillers did not interfere with the polymerization process. Fillers can increase DC by enhancing light scattering and heat retention during curing, which improves polymerization efficiency (41).

Although DMADDM has been shown in previous study to exert minimal or no impact on DC (26). In this study, DMADDM was held constant at 3% across all experimental groups. Thus, the observed differences in DC are attributed primarily to variations in NACP and glass filler content. Overall, incorporation of NACP and glass fillers improved the polymerization efficiency of the orthodontic aligner attachment resin compared to Transbond control and Vitremer control. All experimental groups achieved DC values above the clinically acceptable threshold of 55%, as reported in previous study, supporting their potential for durable clinical performance (42). A higher degree of conversion also correlates with a lower amount of unreacted free monomers, which helps reduce cytotoxicity and improves the longevity of the attachments (43). This suggests that the novel formulation not only offers functional advantages but also supports better clinical biocompatibility.

The antimicrobial results of this study clearly demonstrated the effectiveness of the novel orthodontic aligner attachment resin in reducing *S. mutans* biofilm activity. The incorporation of 3% DMADDM, a quaternary ammonium monomer, played a key role in achieving strong antibacterial action. All experimental groups showed a significant reduction in biofilm CFU counts by approximately a 6-logs, indicating substantial suppression of bacterial colonization. Additionally, biofilm metabolic activity was reduced by over 90%, and lactic acid production decreased by approximately 85%–90%, confirming reduced bacterial viability and acidogenic potential. These findings are consistent with previous studies, which have demonstrated the ability of DMADDM-containing dental resins to disrupt bacterial membranes and reduce biofilm formation (26). The addition of NACP further enhanced the material by providing pH-responsive ion release that may contribute to enamel remineralization and protection. Moreover, the *S. mutans* biofilm counts for Transbond control in this study (∼10 log₁₀ CFU/disk) were similar to the Transbond control reported previously (∼9.2 log₁₀ CFU/disk), confirming the minimal antibacterial effect typical of conventional orthodontic attachment materials. Previous reports have also shown that Vitremer control exhibits limited antibacterial activity, which is consistent with our results (13, 44). The antibacterial effects of this novel orthodontic aligner attachment material highlight its potential for minimize white-spot lesions and improve overall oral health during clear aligner therapy.

Previous studies have evaluated the cytotoxicity of UV-cured resins containing different DMADDM concentrations (16). These compositions exhibited strong antibacterial activity, but also demonstrated excellent cell viability in human gingival fibroblasts (HGF) and dental pulp stem cells (DPSCs) relative to commercial controls. In addition, studies have shown that resin material containing NACP and DMADDM result in lower inflammatory responses and promote tertiary dentin formation in a rat tooth cavity model (45). However, further *in vivo* studies are necessary to comprehensively evaluate the biocompatibility and long-term safety.

Despite these promising outcomes, several limitations should be considered when interpreting the results and designing future studies. First, although the experimental resin was formulated with nano-amorphous calcium phosphate (NACP) to enable sustained calcium and phosphate ion release, its actual remineralization effect on enamel surfaces was not directly assessed in this study. Future investigations will therefore include enamel microhardness and remineralization assays to confirm its protective capability against demineralization and white-spot lesion formation. Second, the antibacterial performance was evaluated using a single-species *S. mutans* biofilm model. While *S. mutans* is a well-established cariogenic bacterium, it does not reflect the complexity of natural polymicrobial oral biofilms, which involve interspecies interactions, and varied metabolic states that can increase resistance to antibacterial agents. Consequently, the antibacterial effects demonstrated here may differ when tested against mature polymicrobial biofilms. Future research will incorporate polymicrobial models derived from human saliva to better simulate clinical conditions and assess long-term biofilm control. Third, this study primarily focused on the material's mechanical strength and antimicrobial properties; other important aspects such as long-term aging, wear resistance, and biosafety need to be investigated in further study. Previous studies from our group showed that UV resins containing 3% DMADDM demonstrate excellent cytocompatibility, comparable to commercial materials, when tested using human gingival fibroblasts and dental pulp stem cells (16). Nonetheless, biocompatibility study on the new attachment composite for clear aligner therapy of the present study will be required to ensure biosafety. Addressing these aspects will strengthen the translational relevance of the new attachment composite for clear aligner therapy in clinical applications.

## 5 Conclusion

This study developed a novel antibacterial attachment composite for clear aligner therapy by incorporating DMADDM, NACP, and glass fillers into a UDMA/TEGDVBE resin. This bioactive orthodontic clear aligner composite attachment demonstrated excellent mechanical properties, a high degree of conversion, and potent antibacterial activity. It substantially reduced *S. mutans* biofilm formation, metabolic activity, and acid production. The addition of NACP may contribute to enamel remineralization. Therefore, the new orthodontic aligner attachment composite is promising to minimize white-spot lesions and enhance preventive care during orthodontic treatment with clear aligners.

## Data Availability

The raw data supporting the conclusions of this article will be made available by the authors, without undue reservation.
